# Prevalence Rate, Probable Causes, and Perinatal Outcomes in Women With Oligohydramnios in Labor

**DOI:** 10.7759/cureus.61290

**Published:** 2024-05-29

**Authors:** Shreen S Mohammed, Amal A Ahmed

**Affiliations:** 1 Obstetrics and Gynaecology, Duhok Maternity Teaching Hospital, Duhok, IRQ

**Keywords:** mortality, oligohydramnios, hospitalization, complication, labor

## Abstract

Background: Oligohydramnios is a common clinical condition among pregnant women. It has direct effects on maternal and fetal outcomes. The related complications in women with oligohydramnios have not been determined sufficiently in developing countries yet. This study aimed to determine the prevalence, portable causes, and perinatal outcomes among women with oligohydramnios.

Methodology: In this follow-up cross-sectional study, pregnant women with oligohydramnios were monitored until delivery at the Duhok Maternity Teaching Hospital to assess the outcomes of oligohydramnios between May 2022 and May 2023. The patients of this study were included through a purposive sampling technique.

Results: More than half (121, 60.5%) of the participants were aged between 20 and 29 years. The gravidity range of the studied pregnant women was 1-9. A total of 156 (78.0%) women have had a history of one to three pregnancies. More than half (102, 51.0%) of studied pregnant women were nulliparous. The study found that 92 (46.0%) had preterm births and the remaining women had term births (108, 54.0%). The most common medical problems among studied pregnant women with oligohydramnios were hypertension (14, 7.0%) and hyperthyroidism (7, 3.5%), and the most common surgical problems were cesarean section (30, 15.0%) and appendectomy (14, 7.0%). The highest prevalence of AFI was 3 cm (84, 42.0%) and 4 cm (82, 41.0%) followed by 2 cm (34, 17.0%). The most prevalent ultrasound finding was fetal hypoxia in 41 (21.5%) women. Most patients' Doppler ultrasound was normal (150, 75.0%).

The study found that 187 (93.5%) pregnancies ended with the birth of live babies and 6.5% (13) of the newborns died. Only three newborn babies (1.5%) reported with low Apgar scores. Low birth weight was reported in 56 (28.0%) newborn babies. The proportion of cesarean sections among women was 94 (47.0%). A total of 117 (58.5%) newborn babies were admitted to the neonatal intensive care unit (NICU) for intensive care.

Conclusions: This study showed that a considerable percentage of women with oligohydramnios were older; had higher gravida, parity, and preterm pregnancies; and had previous cesarean section scars. The most common fetal complications were fetal hypoxia, death, low birth weight, and NICU admission. The most common maternal complication was cesarean section.

## Introduction

Amniotic fluid (AF) is clear and watery and contained within the amniotic sac. It is believed to originate either from maternal plasma via the chorioamnion or from fetal plasma through the permeable fetal skin before keratinization in the third trimester. Amniotic fluid volume (AFV) is maintained through a balance between fetal fluid production, primarily from lung fluid and urine, and fluid resorption through fetal swallowing and flow in the second and third trimesters. This balance occurs across the fetal membranes into the uterus [1]. The volume of AF varies depending on gestational age, typically increasing from around 50 mL at 12 weeks to approximately 400 mL by mid-pregnancy, and then decreasing to about 100 mL at term, with further reduction post-term [2,3]. Evaluation of AFV is a crucial aspect of each sonogram or obstetric screening and is also integral to antepartum fetal surveillance [4,5].

Oligohydramnios is a common clinical condition among pregnant women, playing a significant role in obstetrics due to its direct impact on maternal and fetal outcomes. It can be diagnosed through routine follow-up appointments and may also be detected accidentally in women who do not attend regular antenatal care during pregnancy [6].

Oligohydramnios marks a decline in the AFV < expected volume for gestational age. Polyhydramnios is defined as when AFV is greater than expected for gestational age. Oligohydramnios is defined as an amniotic fluid index (AFI) <5th centile for gestation, or AFI ≤ 5 cm, or the single deepest pocket (SDP) < 2 cm [7]. According to the American College of Obstetricians and Gynecologists practice bulletins, an AFI between 5 and 24 cm is considered normal.

Oligohydramnios, a condition marked by reduced AF levels, can arise in both uncomplicated and complicated pregnancies. Studies show that oligohydramnios is associated with an increased risk of several adverse outcomes, such as neonatal intensive care unit (NICU) admission, meconium-stained AF, meconium aspiration syndrome (MAS), cesarean delivery, lower five-minute Apgar scores, umbilical cord blood pH levels below 7.10, low birth weight (LBW), and respiratory distress syndrome [4,9,10].

Oligohydramnios can result from various causes, including idiopathic origins and maternal factors like preeclampsia or chronic hypertension. Fetal factors such as congenital anomalies, post-term pregnancy, ruptured membranes, and intrauterine growth restriction (IUGR) due to placental insufficiency also contribute to its development. Additionally, placental issues like abruptio placentae and twin-twin transfusion syndrome can lead to oligohydramnios [11].

Complications related to oligohydramnios vary depending on the gestational age at diagnosis. When oligohydramnios is diagnosed in the early stages of pregnancy, it is associated with more serious outcomes, such as birth defects, pulmonary hypoplasia, and an increased risk of stillbirth or miscarriage.

In later stages of pregnancy, oligohydramnios are linked to IUGR, preterm birth, intrauterine fetal demise (IUFD), intrapartum fetal distress, and birth asphyxia. Diagnosis of oligohydramnios during labor is associated with adverse outcomes such as cord compression, meconium-stained fluid, abnormal fetal heart rate patterns, increased likelihood of operative interventions, higher risk of cesarean delivery, lower Apgar scores at birth, higher rates of NICU admission, and neonatal mortality [9,12,13].

As defined by the AFI, oligohydramnios are diagnosed by ultrasound in the second or third trimester [14]. The rate of oligohydramnios is between 0.5% and 8% in pregnant women [15]. The rate of oligohydramnios is elevated in 37% of pregnancies with fatal anomalies and is even higher in other pregnancy complications [16]. Given that ultrasound is not available in clinical settings in many low- and middle-income countries (LMICs), the associated complications in women with oligohydramnios have not been determined sufficiently in LMICs [6]. 

This study aimed to determine the prevalence, portable causes, and perinatal outcomes among pregnancies with oligohydramnios.

The objectives of the study were to describe the sociodemographic and obstetrical characteristics of women with oligohydramnios in labor and determine the maternal complications and morbidity among women with oligohydramnios in labor. 

We hypothesized that the patients with oligohydramnios have higher rates of maternal and neonatal adverse outcomes.

## Materials and methods

Study design and sampling

In this prospective cross-sectional study, pregnant women diagnosed with oligohydramnios were followed up until delivery at the Duhok Maternity Teaching Hospital from May 1, 2022, to May 1, 2023. Women attending the hospital were screened for eligibility criteria, and those diagnosed with oligohydramnios who met these criteria were enrolled in the study. These participants were monitored through delivery to assess the outcomes associated with oligohydramnios. Patients were selected using a purposive sampling technique.

The setting of the study

Data for this study were collected from the Duhok Maternity Teaching Hospital in Duhok City, the sole medical facility providing diagnostic and therapeutic services for pregnant women and prenatal care in the region. The data collection spanned one year, from May 2022 to May 2023. Efforts were made to include all eligible cases of oligohydramnios in the study. The Duhok Maternity Teaching Hospital is one of the main medical settings for diagnostic and therapeutic services to pregnant women in Duhok Governorate. Duhok Governorate is one of the four governorates within the Kurdistan Region of Iraq. It is located in the northern part of Iraq and borders Turkey, Syria, and other Iraqi governorates.

Inclusion criteria

The following inclusion criteria were applied to the patients.

· Proven cases of oligohydramnios by ultrasonography with AFI < 5 cm

· Gestational age > 25 weeks

· Singleton pregnancy

Exclusion criteria

The following exclusion criteria were applied to the patients.

· AFI > 6 cm

· Multiple pregnancies

· Patients having major respiratory and cardiovascular diseases or other abdominal conditions

Statistical analyses

In this study, patients’ demographic and medical characteristics were retrieved from their medical records following approval from the relevant health ethics committee. Patient outcomes were obtained from medical records post-delivery. The outcomes of patients with oligohydramnios were expressed in numbers and percentages. Statistical analyses were conducted using IBM SPSS Statistics for Windows, Version 25.0 (IBM Corp., Armonk, NY), released in 2017 (https://www.ibm.com/products/spss-statistics).

Data collection and measurement criteria

The data required for this study were collected from the medical records of the patients and recorded in a predesigned questionnaire. This questionnaire comprised three parts. The first part contained demographic and general information, including age and occupation. The second part documented medical and obstetric information such as gravidity, parity, previous abortion, gestational age, past medical conditions, and past surgical conditions. Finally, the third part of the questionnaire recorded maternal and neonatal outcomes.

In detail, the outcomes of the study were measured as follows: The AFI was measured in centimeters and categorized as AFI of 2, 3, and 4 cm. Doppler ultrasound findings were recorded as normal, fetal hypoxia, intrauterine growth retardation, low-level placenta, and placental insufficiency. Delivery outcomes were recorded as alive or dead babies. Apgar scores, measured between 0 and 10, were categorized as 0-3 (low Apgar score), 4-6 (intermediate Apgar score), and 7-10 (normal Apgar score). Birth weight, documented in grams, was categorized as normal birth weight (2,501-4,000 g), LBW (≤2,500 g), and macrosomia weight (>4,000 g). Additionally, the types of delivery were recorded as normal vaginal delivery and cesarean section. NICU admission and gross congenital anomalies at birth were recorded as yes or no.

Ethical confiscations

The protocol for this study received ethical approval from the local health committee in Duhok City. Since the necessary information was collected from patients' medical records, verbal and written consent forms were not required. However, strict measures were taken to ensure the confidentiality of patients' personal information throughout the study.

Furthermore, the study protocol was approved by the Kurdistan Board for Medical Specializations and was officially registered as number 786 on March 17, 2023.

## Results

The age range of the participants in the study spanned from 17 to 42 years, with a mean age ± standard deviation (SD) of 27.8 ± 5.5 years. A majority of the participants (121, 60.5%) fell within the age range of 20 to 29 years, while those under 20 years and over 40 years constituted only 10 (5.0%) and 6 (3.0%), respectively. The vast majority of pregnant women (180, 90.0%) who took part in the study were housewives. Regarding gravidity, the range among the studied pregnant women was from 1 to 9, with a mean gravidity ± SD of 2.33 ± 1.72. The majority (156, 78.0%) reported a history of one to three pregnancies, including the current one. Only a small percentage (5, 2.5%) had a history of seven or more pregnancies. The parity range among the participants was from 0 to 7, with a mean parity ± SD of 1.16 ± 1.52. More than half (102, 51.0%) of the studied pregnant women were nulliparous, with only one woman (0.5%) having given birth to seven children. Out of the total 200 pregnant women studied, 162 (81.0%) had no history of previous abortion, while multiple abortions (three or more spontaneous abortions before 20-28 weeks of gestation) were reported in only 2 (1.0%) pregnant women.

All pregnant women with a gestational age of 26-42 weeks or beyond were included in the study, while those with a gestational age of less than 26 weeks and those with chronic disorders were excluded. The mean gestational age of the participants was 37 weeks. Regarding birth outcomes, the study found that 92 (46.0%) had pre-term births, while the remaining 108 (54.0%) had term births, with no cases of post-term birth observed (Table 1).

**Table 1:** Sociodemographic characteristics, gravidity, parity and previous abortion, and gestational age of the studied pregnant women with oligohydramnios at Duhok Maternity Hospital (n = 200).

Sociodemographic characteristics (*n *= 200)	*n* (%)
Age group (17-42 years)	
15-19 years	10 (5.0)
20-29 years	121 (60.5)
30-39 years	63 (31.5)
40 years or older	6 (3.0)
Occupation	
Housewife	180 (90.0)
Employed	20 (10)
Gravidity	
Gravida 1-3	156 (78.0)
Gravida 4-6	39 (19.5)
Gravida 7 or more	5 (2.5)
Parity	
Para 0	102 (51.0)
Para 1-3	76 (38.0)
Para 4-6	21 (10.5)
Para 7 or more	1 (0.5)
Previous abortion	
No abortion	162 (81.0)
Abortion 1-2	36 (18.0)
Three or more abortions	2 (1.0)
Gestational age (weeks)	
Preterm birth (< 37 weeks)	92 (46.0)
Term birth (37-41 weeks)	108 (54.0)
Post-term birth (42 weeks or beyond)	0 (0.0)

The predominant medical issues identified among the pregnant women studied with oligohydramnios were hypertension (7.0%) and hyperthyroidism (3.5%). The majority of patients had no prior medical conditions (172, 86.0%). Regarding surgical history, the most frequently reported problems among these women were cesarean section (15.0%) and appendectomy (7.0%; Table 2).

**Table 2:** Past medical conditions in pregnant women with oligohydramnios at Duhok Maternity Hospital (n = 200).

Past medical and surgical conditions (*n *= 200)	*n* (%)
Past medical conditions	
No past medical conditions	172 (86.0)
Hypertension	14 (7.0)
Hyperthyroidism	7 (3.5)
Gestational diabetes	2 (1.0)
B-thalassemia trait	1 (0.5)
Preeclampsia	1 (0.5)
Pregnancy-induced hypertension	1 (0.5)
Polycystic kidney disease	1 (0.5)
Hydatid cyst of liver	1 (0.5)
Past surgical conditions	
Negative	150 (75.0)
Cesarean section scar	30 (15.0)
Appendectomy	14 (7.0)
Cholecystectomy	3 (1.5)
Thyroidectomy	1 (0.5)
Hemorrhoidectomy	1 (0.5)
Tonsillectomy	(0.5)

This study focused on AFI measurements of less than 5 cm. The highest prevalence of AFI was observed at 3 cm (84, 42.0%) and 4 cm (82, 41.0%). The least prevalent AFI recorded among the studied women was at 2 cm (17.0%). Among the ultrasound findings, fetal hypoxia was the most commonly reported among pregnant women with oligohydramnios, affecting 41 (21.5%) individuals. Notably, the Doppler ultrasound results were normal for most patients (150, 75.0%; Table 3).

**Table 3:** Amniotic fluid index (AFI) and Doppler ultrasound among studied pregnant women at Duhok Maternity Hospital (n = 200).

AFI and Doppler ultrasound (*n *= 200)	*n* (%)
AFI (cm)	
2	34 (17.0)
3	84 (42.0)
4	82 (41.0)
Doppler ultrasound findings	
Normal	150 (75.0)
Fetal hypoxia	43 (21.5)
Intrauterine growth retardation	2 (1.0)
Low-level placenta	1 (0.5)
Placental insufficiency	2 (1.0)

More than half (54.5%) of the newborn babies were male. Among the total of 200 pregnancies studied, 187 (93.5%) resulted in the birth of live babies, while 13 (6.5%) newborns did not survive. Only three newborn babies (1.5%) had a low Apgar score out of the total 200 deliveries. The birth weight range among the newborn babies was from 800 to 4,600 g, with a mean birth weight ± SD of 2,787 ± 0.71 g. LBW was observed in 56 (28.0%) newborns. Cesarean sections accounted for 94 (47.0%) deliveries among the studied women. Additionally, 117 (58.5%) newborn babies were admitted to the NICU for intensive care. Only two newborn babies (1%) were reported to have gross congenital anomalies at birth (Table 4).

**Table 4:** Pregnancy outcomes of studied pregnant women with oligohydramnios at Duhok Maternity Hospital (n = 200). NICU, neonatal intensive care unit; AFI, amniotic fluid index

Pregnancy outcomes	*n* (%)
Delivery outcome	
Alive	187 (93.5)
Dead	13 (6.5)
Apgar score (0-10)	
0-3 (Low Apgar score)	3 (1.5)
4-6 (Intermediate Apgar score)	10 (5.0)
7-10 (Normal Apgar score)	187 (93.5)
Birth weight (g)	
2,501-4,000 (Normal birth weight)	141 (70.5)
≤2,500 (Low birth weight)	56 (28.0)
>4,000 (Macrosomia weight)	3 (1.5)
Type of delivery	
Normal vaginal delivery	106 (53.0)
Cesarean section	94 (47.0)
NICU	
Yes	117 (58.5)
No	83 (41.5)
Gross congenital anomalies at birth	
Yes	2 (1.0)
No	198 (99.0)

## Discussion

This study revealed a notable proportion of women with oligohydramnios who were older and had higher gravidity, parity, and incidence of preterm pregnancies. Moreover, a significant percentage of these women had previous cesarean section scars. The most prevalent fetal complications observed were fetal hypoxia, mortality, LBW, and admission to the NICU. Among maternal complications, cesarean section was the most common.

In the Kurdistan Region and Iraq, limited research has been conducted on oligohydramnios and their outcomes. Ibrahim and Zween conducted a retrospective study involving 150 patients diagnosed with oligohydramnios and 150 patients with normal AFI in Erbil [17]. Consistent with our findings, their study revealed that patients with oligohydramnios were more inclined to have higher parity and lower gestational age. Additionally, individuals with oligohydramnios exhibited higher rates of cesarean section compared to the normal group. Oligohydramnios was also linked to increased rates of intrauterine growth retardation and LBW. Another study conducted in Duhok City reported similar findings [18]. In agreement with our study, they reported that the oligohydramnios were associated with a higher rate of hypertension and gestational diabetes.

Similar findings have been reported in other studies across the world [19,20]. In addition, the review studies have reported similar findings. For example, a review study included the related studies from 1980 to 2015. They included 15 clinical trials in the study [21]. Their findings indicated that patients with oligohydramnios had notably higher rates of infants with meconium aspiration syndrome (relative risk [RR] 2.83; 95% confidence interval [CI] 1.38-5.77), cesarean section for fetal distress (RR 2.16; 95% CI 1.64-2.85), and NICU admission (RR 1.71; 95% CI 1.20-2.42) compared to those with normal AFI. Moreover, patients with oligohydramnios and comorbidities exhibited significantly higher rates of LBW (RR 2.35; 95% CI 1.27-4.34). However, no significant differences were observed in the incidence of five-minute Apgar score <7, NICU admission, meconium-stained amniotic fluid, and cesarean delivery for fetal distress between patients with oligohydramnios and those with normal AFI.

The perinatal effects of oligohydramnios on some outcomes such as birth weight, rate of cesarean section, and Apgar scores are not consistent in the literature. Locatelli et al. [22] reported that the oligohydramnios pertained to the higher risk of LBW. However, other studies did not report a significant difference in LBW between the oligohydramnios and control groups [23-25].

According to our findings, fetal hypoxia was the most common complication among fetuses of women with oligohydramnios in this study. The literature has documented the impact of maternal oligohydramnios on the risk of respiratory illness among children. For instance, Biard et al. [26] found that 50% of children with a history of oligohydramnios and lower urinary tract obstruction experienced long-term respiratory issues. These primarily included asthma and recurrent respiratory tract infections. However, the exact mechanism underlying this effect remains unclear, as it is not feasible to directly assess lung tissue in infants and children.

Neonates with pulmonary hypoplasia typically require prolonged respiratory support, such as oxygen supplementation and mechanical ventilation. The use of mechanical ventilation in infants has been associated with both acute effects and long-term respiratory morbidity.

Oligohydramnios exposure in utero can cause the LBW. LBW (birth weight < 2.5 kg) is associated with hospital admission due to respiratory illness [27]. In this context, Magann et al. [28] found that exposure to oligohydramnios at a low gestational age led to a significant rise in the risk of NICU admission. The oligohydramnios are associated with low neonatal birth weight and higher rates of cesarean section [29].

Our study revealed a high rate of cesarean section among women with oligohydramnios, consistent with findings from existing literature. For instance, a cross-sectional study comparing deliveries and perinatal outcomes in women with and without oligohydramnios reported similar results. This study found that patients with oligohydramnios had higher rates of preexisting gestational diabetes mellitus, fetal growth restriction, obesity, and malpresentation. Additionally, cesarean delivery was significantly more common in pregnancies complicated by oligohydramnios compared to those without oligohydramnios (84.4% vs. 54.7%; *P *< 0.001) [30]. However, the literature has reported controversial findings [31,32].

The oligohydramnios risk factors are premature rupture of membranes, intrauterine growth restriction, and birth defects [29]. Oligohydramnios, characterized by diminished levels of amniotic fluid, can impede the fetus's normal mobility and hinder its growth and development. This condition may potentially result in fetal deformities, compression of the umbilical cord, and, in severe instances, fetal death. In the first trimester, a reduction in amniotic fluid volume is a concerning discovery and may lead to spontaneous miscarriage. However, during the second trimester, the prognosis is heavily influenced by the underlying cause. Borderline to mildly decreased levels of amniotic fluid generally suggest a favorable outcome, whereas severe oligohydramnios is often linked with fetal mortality [33]. In the third trimester, many cases of oligohydramnios have no identifiable cause (idiopathic). Adverse fetal outcomes in these cases are typically linked to factors such as umbilical cord compression, uteroplacental insufficiency, and meconium aspiration [29].

Strengths and limitations of the study

The primary strength of this study lies in our extensive inclusion of a large number of patients diagnosed with oligohydramnios, allowing for robust analysis of this condition. Additionally, we made concerted efforts to encompass a wide range of associated maternal and fetal complications. However, it is important to acknowledge that our study lacked a control group, which could have provided valuable comparative data for a more comprehensive understanding of oligohydramnios and their outcomes.

## Conclusions

This study demonstrated that a significant proportion of women diagnosed with oligohydramnios were older, had higher gravidity and parity, and experienced preterm pregnancies, along with a history of previous cesarean section scars. Among the observed fetal complications, fetal hypoxia, mortality, LBW, and admission to the NICU were the most prevalent. Additionally, cesarean section emerged as the most common maternal complication.
